# “A huge part of my life”: Exploring links between music, medical education, and students’ developing identities as doctors

**DOI:** 10.15694/mep.2018.0000183.1

**Published:** 2018-09-03

**Authors:** Alison Ledger, Viktoria Joynes

**Affiliations:** 1 Leeds Institute of Medical Education; 2 School of Medicine

**Keywords:** music, medicine, medical education, medical students, arts teaching, medical humanities, professional identity development, critical thinking

## Abstract

This article was migrated. The article was marked as recommended.

This paper explores the place of music in the development of future doctors, through the lens of a mixed method, longitudinal evaluation of a two-week music and medicine special studies project for second and third year medical students. Methods of evaluation included a cohort-wide survey (n=147) and individual interviews with students who had undertaken the music and medicine project (n = 4). Analysis of survey responses indicated that music is important to medical students and that many students recognise links between music and medicine. Medical students who undertook the music and medicine project reported benefits for their ongoing development, including changes in the way they understand and use music in their own lives and exposure to career options they had not considered previously. These benefits are discussed in relation to Dennhardt et al.’s (2016) framework of epistemic functions of arts-based teaching in medical education and to wider debates about whether medical humanities teaching should be compulsory or optional. We then propose that there is room for music in medical education within integrated medical humanities teaching, to promote critical thinking and openness to new perspectives. Optional music and medicine study should also be available for medical students who identify as musicians, to support them in the process of developing their identities as doctors.

## Introduction

Links between music and health are well-established in fields such as music psychology, sociology, ethnomusicology, and music therapy ( deNora, 2000; Edwards, 2007; Hargreaves and North, 1997; MacDonald et al *.,* 2012). We know that people listen to music to relax, to achieve and intensify moods, and to accompany and enhance day to day activities ( deNora, 2000; van den Tol and Edwards, 2011). There is also evidence to suggest that sharing music and musical experiences or making music with others leads to feelings of belonging and well-being ( Daykin, 2012; Stige, 2012). Additionally, music therapists around the world use music to assist patients or clients to achieve and/or maintain health and a growing evidence-base supports this practice ( Edwards, 2016).

Within the profession of medicine, interest in music and health has manifested in a variety of ways. There is a long history of doctors’ involvement in bands, orchestras, or choirs, and doctors may use music as a way of coping with the pressures of healthcare work ( Arjmand, 2006; Cook, 2002; Ortega et al., 2011; Smith, 2017). Over the past ten years, there have also been efforts to bring various professionals with an interest in music and medicine together. One notable effort was the establishment of the International Association for Music and Medicine (IAMM), an organisation which aims to promote collaboration between researchers, educators, and practitioners in the fields of music medicine and music therapy, including doctors interested in applying music in their work ( Vatanasapt, 2017). The IAMM journal *Music and Medicine* publishes collaborative studies undertaken by a range of professionals, and mostly includes studies of the benefits of music for patients in medical settings.

Although there are indications that doctors use and are interested in music, there is surprisingly little mention of music in the field of medical education. Despite there being increasing interest in the medical humanities and the use of art in medical teaching, there are only passing mentions of music in recent papers ( Jones et al., 2017; Varpio et al., 2017). Where medical humanities teaching interventions are described in any detail, these tend to focus more on visual arts, narrative, or performance (for examples, see de la Croix, 2011; Kumagai, 2008; Shapiro et al., 2006) and experts have suggested that the medical humanities in medical education are limited by narrow understandings of the value of the arts ( Bleakley, 2017). Watling et al. (2013; 2014) explored similarities and differences in the learning cultures within medicine and music, but their papers focused on what medical educators can learn from teacher-learner relationships and feedback processes in music education.

Our interest in music and medicine stems from Alison’s former career as a music therapist and her efforts to bring this previous experience into her current role as a medical education lecturer and scholar, and Viktoria’s hobby of playing in a band and her academic interest in how involvement with music influences identity development. We designed, delivered, and evaluated a two-week project in music and medicine which was offered to second and third year medical students. A paper by Dennhardt et al. (2016) has been particularly useful to us in understanding both students’ reactions to being involved in this project, and for us in positioning the focus of our teaching. Following a detailed review of literature, Dennhardt and colleagues found three main ways in which the arts are understood in medical education (intrinsic, additive, or curative) and mapped thefoci of arts activity on a continuum of developing expertise, dialogue, and expression and transformation. In reviewing our own teaching in this area, and aligned to the work of Dennhardtet al. (2016), we wished to find out more about the ways medical students understand and make use of music in their everyday lives. We perceived that detailed exploration in this area was important for:

i. developing students’ understandings of the benefits and risks of music for both patients and for themselves;
ii. helping students to develop their identities as doctors;
iii. developing uses of music in medical education, and;
iv. raising awareness of possible career pathways in medicine.

The following section gives a detailed explanation of our teaching, as insufficient detail has been identified as a weakness of previous reports of arts-based interventions in medical education ( Perry et al., 2011).

### The music and medicine project

This paper reports findings from a mixed method, longitudinal study of our two-week music and medicine project, which ran in 2014 and 2015. The music and medicine project was offered to second and third year medical students as a special studies project choice in the two weeks leading up to Christmas. Special studies projects (SSPs) are one of the student selected components of the undergraduate medicine degree (MBChB) at the University of Leeds, introduced to meet General Medical Council expectations with regard to medical school teaching content ( General Medical Council, 1993; 2003; 2009). Special studies projects are intended to offer students experiences outside of mainstream medical education, develop students’ critical reflective skills, and promote enterprise skills such as adaptability, creative thinking and innovation. Each medical student chooses one special studies project per year in years two and three of the medical course.

The music and medicine project was designed to address the theme of music and medicine from multiple disciplinary perspectives, including sociological and therapeutic understandings of music, arts in health, and arts in medical education perspectives. These perspectives arose from the expertise of our teaching team (a medical education academic who was previously a music therapist, a medical education academic with a sociological background, a music teacher undertaking a PhD in the area of Performing Arts Medicine, and a visiting music therapy academic). In developing the teaching content and methods, we wished to expand and challenge students’ understandings of the relationships between music and health; and encourage students to critique common assumptions about music’s positive “effects” (for a published critique see Edwards, 2008). In these respects, the foci of our teaching was intended to be “art as dialogue, expression, and transformation” on Dennhardt et al.’s (2016) continuum of epistemic functions of arts-based teaching in medical education.

Teaching topics included music and identity, research about music and health, benefits and risks of using music in health and social care, health of musicians, an introduction to music therapy as a profession, and arts-based approaches in medical education and research. Teaching involved around 30 hours of small group teaching sessions, seminars, group work, individual tutorials and self-directed learning activities, and students were encouraged to consider their own as well as others’ responses to music throughout. The students’ work was assessed through a critical review of related literature, a written reflection on the project, and a presentation of a music protocol for a healthcare environment. Student numbers were restricted to 10 per cohort to facilitate effective group work and depth of discussion.

## Methods

Data presented here is drawn from a longitudinal evaluation of the music and medicine project, which involved three different methods of data collection. At the outset of the project, we had identified that very little had been written about the use of music in medical teaching, and that a particular weakness of previous research was a lack of long-term follow-up of students ( Perry et al., 2011). The methods used were therefore devised in an attempt to start to address this gap in existing research. Three methods were trialled in an attempt to gather longitudinal data: 1) administration of an online survey of all year 2 and 3 undergraduate medical students before and after the teaching, 2) face to face interviews with music and medicine students one year after the project, and 3) collection of entries in an electronic diary which captured music and medicine students’ ongoing thoughts and experiences related to the music and medicine project.

A link for the survey was sent to all students in years two and three of the medical course in 2015-2016 at two time-points, once prior to the music and medicine teaching period, and once afterwards, in an attempt to establish any immediate differences between the cohorts choosing to study music and medicine, and those who did not choose it. Out of a total number of 506 second and third year medical students, 88 students took part at survey time-point one and 59 students took part at survey time-point two. We asked students at both survey points whether they had already, or intended to apply for, the music and medicine SSP. The survey instrument was the same each time and used a combination of Likert-scale and open-ended questions to establish when students were listening to and using music in their own daily lives, to ascertain their thoughts on the contribution of music to health and wellbeing, and to give an indication as to why (or not) they were interested in the music and medicine project. Participation in the survey was voluntary and entry into a prize draw to win a £25 Amazon voucher was offered as an incentive to take part in the survey at each time-point. Survey results were collated into tables and explored for trends in the data; open ended questions were categorised by each author independently and then discussed for agreement of final themes.

Students who undertook the music and medicine project in either 2014 or 2015 were invited to take part in follow-up interviews about their experiences of the project and continuing development, one year after the project had ended. Four out of twenty music and medicine students volunteered to take part in follow-up interviews, two from the 2014 cohort and two from the 2015 cohort. During the interviews, students were asked what they most remembered about the project and what they had found most valuable in their ongoing learning in the subsequent year. At the end of the interview, students re-read the reflections they had submitted for assessment and commented on whether their reflections remained relevant. As we were heavily involved in developing and leading the teaching for the music and medicine project, an independent colleague (Shelley Fielden) joined the project team to undertake the interviews to maximise authenticity of responses. Shelley audio-recorded the interviews and transcribed them in full; we then used the transcripts to complete the analysis for this paper. Interview transcripts were read independently and main themes were identified. After discussion, we felt the interview data would make powerful case studies of the impact the music and medicine project had on the participants as medical students; thus the data we have chosen to present is in case study form.

A third method involved encouraging participants in the music and medicine project to keep an online electronic diary of their interactions with music. However, only two students volunteered to participate through this method and made only a handful of entries in the year following the project (despite prompts from the research team). These diary entries confirmed our interpretations of the other data sources, rather than contributing new findings. We have therefore decided to focus on the survey and interview data in this paper.

Ethical approval for this research was secured prior to data collection from the University of Leeds School of Medicine research ethics committee.

## Results/Analysis

Findings from the survey and interviews are reported separately below.

### Medical students’ perceptions of music and medicine

**Figure 1.**
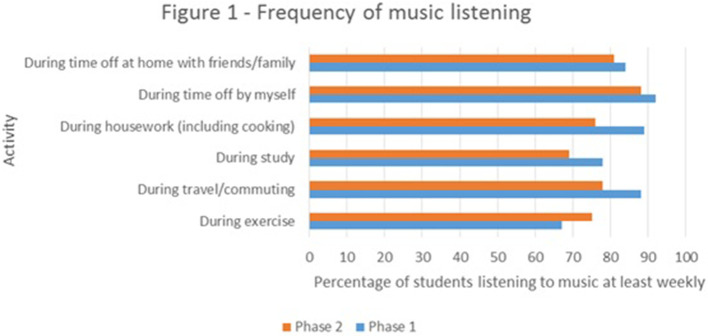
Frequency of music listening

The survey results indicated that a majority of medical students were listening to music at least once a week during time off at home and during activities such as housework and commuting (see figure 1). In terms of active engagement in music, they also appeared to be a high achieving cohort, with at least 44% of respondents in each survey having achieved a UK Grade 5 (or equivalent) musical examination or higher. Of most interest was the fact that there were no discernible differences between the musical engagement of the cohort who took the music and medicine SSP and the rest of the student respondents. Furthermore, analysis of the open-ended survey results revealed 4 distinct themes within the responses given by students to the question of why, or why they would not, consider undertaking the music and medicine SSP. These are worth drawing out here due to their implications for others who may be interested in introducing such content into medical curricula.

*Theme 1: Misconceptions of what music and medicine study would involve*

The music and medicine project was intended to be open to any second or third year medical student, regardless of their level of musical ability or experience. It was therefore surprising that survey respondents held misconceptions around what studying music and medicine would entail, or what skills were required in order to find the project useful. Many students mentioned that they were not musical, or that they would not be able to engage fully as they would not be able to perform on an instrument.

*Don’t feel I am musical enough (P1-28)*

*I’m not sure I’d be good at it - I find technical music things difficult (P1-38)*

Further misconceptions about the course were revealed by students who felt that it drew too heavily on a certain style of music in which they would not be interested:

*The course looked too wide access and general (P2-19)*

*..I felt it was oriented toward singing / popular music, and, although I would still enjoy it, I prefer to play classical music (P2-31)*

Such perceptions were surprising because there were no defined pre-requisites of the project in terms of musical ability and students were invited to come and share music of any style that was meaningful for them. The perception that music and medicine was not as ‘open for all’ as we had intended it to be was therefore an interesting one.

*Theme 2: A perception that there was ‘nothing new to learn’ on the topic*

Some students suggested that having previously studied elements of music, or looked into the relationship between music and medicine, there was nothing new for them to learn in a music and medicine SSP.

*I feel I already understand a lot about music and its role in medicine and therefore felt I wanted to learn something entirely new (P2-13)*

*Have had involvement with the effect of music and medicine before at home in a voluntary group and want to use [my] SSP to expand my knowledge of new things (P2-25)*

As educators interested in introducing music into medical education we chose to view such responses as heartening ones; these students were not averse to the ideas we were seeking to introduce, but were seeking to broaden their opportunities further which is one of the positive features of SSPs.

*Theme 3: Not interested in music and medicine as a topic / more interested in other topics*

Some students highlighted that they did not pick the music and medicine SSP as it was not a topic that they were interested in. One student expressed a distinct perspective, stating:

*I enjoy music as a hobby and would not like to associate it with medicine (P1-30)*

Also within this category was a subset of students who stated they would have potentially been interested in the topic but, given the limited number of choices they were able to make, selected options they were more interested in or chose to explore something new as part of the SSP programme instead.

*I considered it strongly because I love music, however in the end I wanted to try something new so went for tai chi (P1-40)*

*I had more interest in another project which took precedence (P1-26)*

Such responses again suggest an openness from medical students to explore study options that are less ‘typical’ of medical education, but they problematize the viability of introducing music and medicine style topics to the whole cohort if some will simply not be interested in engaging with it.

*Theme 4: An interest in the therapeutic potential of medicine*

For those students who did express interest in taking music and medicine as an SSP (some of whom did then take it, and some of whom did not), they often reported that this interest related to the therapeutic potential of music and their interest in exploring this further, either to influence their practice as a future doctor, or to understand it further for themselves.

*..I tend to play music based on my mood and listen to lots of different genres of music and wanted to explore the whole topic in more depth and learn new things (P1-51, before starting the spirituality SSP, music and medicine was second choice)*

*..I chose it because music has always been a strong influence in my life and I have spent a lot of time working in music as well as playing/listening for enjoyment. I think that it has a huge scope medically. (P1-36, before music and medicine SSP)*

Consistent with a study of musicians with lay or professional interests in health ( McLellan et al., 2013), many survey respondents perceived that music was “beneficial for health”. Although some responses indicated that music may not be of interest to all, there remained a striking number of respondents who seemed captivated by relationships between music and medicine and could see music as being relevant to their medical education.

### Experiences of music and medicine students

The four follow-up interviews indicated that the music and medicine project had lasting impacts on students, both intended and not anticipated. For the purposes of brevity, three case studies have been selected to show the range of responses which students communicated. The fourth case was no less powerful, but replicated experiences conveyed in the following three case studies.

*Case Study 1: Jennifer, thinking differently about music*

Jennifer was a third year student at the time of the SSP and intercalating in medical education at the time of interview. She described herself as a musician, “but not a very good one”. She played in two bands, the medics big band and the university-wide concert band, in order to mix with a range of people and develop friendships with “normal” people outside of medicine. Jennifer also described listening to recorded music frequently, particularly when on the bus and while studying.

When asked what most stood out for her from the music and medicine project, Jennifer recalled a session in which students shared pieces of music that they could associate with an aspect of clinical placement. She explained how she still hears one of these songs now and is always reminded of the student who chose it and the particular student group. The other session that was most memorable for Jennifer was the one led by the visiting music therapy academic (Simon Gilbertson), as it challenged her to go beyond her “comfort zone”.

*I think he had such a very different outlook to life than I personally have, I think I have very.. probably a scientific view really, given like my interests.. and he was just very literally ‘outside the box’.. it was something so different to how I’d ever experienced like teaching before.. it just kind of blew me away really..*

The sessions around benefits and risks of music for health further challenged Jennifer to be more thoughtful about her music choices. She said she had never before considered that music could be unhelpful, and spoke of times since when she had decided to turn music off because she realised it was distracting (e.g. when revising or writing an essay). Jennifer also recalled a time when she had made a conscious decision to attend the medics big band rehearsal even though her peers had decided to stay home to revise for their exams.

*I’d thought that actually no, for me, this is the best way to do it.. I knew that I wouldn’t do any really productive revision.. the [music and medicine] project was probably, maybe was in the back of my mind then, because I was thinking you know, this is useful for me, I shouldn’t feel guilty about taking the time out.*

*Case Study 2: Johnny, using music for well-being*

Johnny was a third year student at the time of the SSP and intercalating in medical education at the time of interview. He had always sung with family, but felt that the music and medicine project had made him “reassess the importance of music in my life”. Since the project, he had started playing the guitar and the ukulele, was listening to new music more, and had taken a greater interest in music journalism. Johnny understood his renewed interest in music as a “means of unwinding” and perceived that reading music magazines encouraged him to consider perspectives different to his own. For Johnny, the “subjective” nature of music preference was reinforced by the session on music and identity and the one in which students shared music with the group. He particularly recounted how listening to other students’ music encouraged him to listen to genres of music he would not normally listen to (e.g. classical) and to recognise the benefits music had for different people.

*It was clear that there wasn’t any sort of, um, absolute trend, everyone’s different and I think using music to highlight that really um, really drove it home and it’s made me judge people less by their types of music and perhaps made me more open-minded myself about the type of music I listen to.*

Johnny also drew attention to the value of discussing topics which he had not thought about previously, such as the use of music in regulating emotions, using music as therapy, and health problems experienced by performing musicians.

*I’d never really thought of music-related, um, injuries, from kind of repetitive strain from playing the violin, to just the kind of laryngitis from, from singing, so I think that was quite um, quite eye opening*

Like Jennifer, Johnny strongly recalled the session with Simon Gilbertson and explained how the session had taken on a new relevance since he had started his own medical humanities research project. One particular quote from Simon stood out for Johnny - “If something is difficult to discover but meaningful, then it has the necessary qualities for research”.

*Case Study 3: Tracy, music and medicine as career-changing*

Tracy was in second year when she undertook the music and medicine SSP and was a third year medical student at the time of interview. Tracy was a singer-songwriter and performing musician, who toured with her bands around medical school commitments. She saw the music and medicine SSP as an exciting opportunity to bring two separate parts of her life together.

*..’cause music’s a huge part of my life and has been.. since I was a teenager.. I just thought.. I would love to learn, how I can integrate the two.*

Of the students interviewed, Tracy provided the most direct examples of ways that the music and medicine project had influenced her interactions on clinical placement. She described talking to patients about music, questioning the meaning of patients’ preferred music, looking for evidence of arts therapies at placement sites, using music to reflect on her placement experiences, and even suggesting music therapy for a patient with ongoing depression on her general practice placement.

*I’d feel comfortable suggesting it [music therapy] to, to GPs now, or to anyone, like any doctor I was around like I wouldn’t feel like embarrassed or uncomfortable suggesting it and I feel like I have better knowledge of it and like when it could be used.. I can back up why.*

The session on the health of musicians was especially influential for Tracy in thinking about the type of doctor she wished to be. Though she had entered medicine wanting to practice emergency medicine, she now wanted to develop a career in performing arts medicine. Since the project, Tracy had become a member of the British and American associations for performing arts medicine, attended a performing arts medicine conference in the United States, developed performing arts medicine contacts, and was an avid reader of the journal Medical Problems of Performing Artists. She had explored the possibility of undertaking a Masters in Performing Arts Medicine, brought her interest in music and medicine into her SSP the following year (spirituality), and was planning to develop research about musicians’ mental health for her final year project. For the first time, Tracy had considered a career in primary care attractive, as she felt she could improve musicians’ access to healthcare by becoming a GP herself.

*I hadn’t considered it at all, as a career.. I didn’t know I could do it.. I’d probably still be completely in the dark, yeah if it hadn’t been for this project.. it’s had such a huge impact on everything I do now like to do with medicine and like everything I’m working towards, it’s just completely changed, my direction..*

Tracy also emphasized the value of the project in motivating her to continue her university studies (the undergraduate medical course was her second degree).

*Hopefully if I work hard at it, in the future, I can like use 2 things that I’m obviously really passionate about in 1 area and it’s made it a lot easier I think to study.*

## Discussion

Results from our survey suggested that second and third year medical students at our university had high levels of engagement and experience with music. Furthermore, one of the main reasons cited for not choosing the music and medicine special studies project was that students already engaged in music outside of medicine or were already aware of the potential health benefits of music. As noted by Dennhardt et al. (2016), much of the literature on arts interventions assumes that the arts are new to students, or should be introduced to counteract weaknesses in medicine such as a perceived lack of humanity or holistic care. Previous studies of the arts in medical education have suggested that the arts can produce better or more empathic doctors ( Bleakley, 2015; Perry et al., 2011). Our study suggests that music is not necessarily something “additive” or “curative”, but already an intrinsic part of our students’ identities and everyday lives. As students are frequently asked to reflect on their activities outside of medicine as part of their personal statement to gain admission to medicine courses, our context is likely to be similar to other medical schools, at least within the UK.

The extent to which students wanted to bring music into their medical practice and identities varied. Most survey participants expressed a desire to explore how music and medicine could be brought together. However, we were surprised to learn that one survey participant ‘would not like to associate it with medicine’. This statement raised multiple questions for us, which could also apply to other medical humanities topics. Do we, as educators, think that this wish should be respected? Or should all students be encouraged to consider relationships between music and medicine? What does this statement say about this student? Is there something unique about music which some students want to keep private and separate to medicine? The interviews were helpful to us in considering these questions further.

Follow-up interviews with students who did undertake the music and medicine project revealed educational benefits that were both intended and unexpected. Interviews with Jennifer and Johnny indicated that they had gained a more critical understanding of relationships between music and health, but we were pleasantly surprised to learn that the students also perceived changes in their behaviour since the project. Jennifer suggested that she had become more discerning about the use of music in her everyday life, whereas the music and medicine project seemed to be a stimulus for Johnny to re-engage with music as a way of taking a break from the pressures of medicine. Tracy was taking every opportunity to bring music into her medical practice and had taken steps to develop a career in performing arts medicine. This was a career she had not considered before the music and medicine project. It has also been interesting to note that three of the twenty students who took the music and medicine SSP in 2014 or 2015 went on to undertake research on related topics.

In considering Dennhardt et al.’s (2016) framework, these benefits may be best understood as “arts as expression and transformation”. The music and medicine project seemed to promote personal development, one of the intended learning outcomes of the SSPs at our university. Findings from our interviews suggest that a particular focus on music may lead to changes in the ways students use music for their own health and wellbeing. Additionally, we noted the potential for music and medicine study to allow students with a strong background in music to bring two seemingly separate aspects of their identity together. In the past decade, authors have drawn attention to the potential for medical students to experience identity dissonance in the process of developing their identities as doctors ( Joseph et al., 2017; Monrouxe, 2010). Students whose personal identities and values are different to those expected of a doctor may experience stress, uncertainty, and feel like they do not belong in medical school ( Joseph et al., 2017). It is important for us as educators to understand how such dissonance can impact upon the lives of our students and potentially their success at medical school. Music and medicine study may allow students to explore ways that their musical selves fit with current understandings of medical practice, but also promote awareness of any sources of identity dissonance. Greater awareness of identity dissonance could lead students to recognise a need for support in their process of professional identity development, allow them to achieve more of a sense of ‘belonging’, or perhaps even enable them to question established cultures in medicine.

Bleakley (2015; 2017) proposed that the arts in medical education should be disruptive and has criticised interventions in which arts are merely used “in service” to medicine. We did not necessarily observe our music and medicine project to be disruptive to the extent that Bleakley proposed, but students did share examples of ways that their thinking had been challenged. A particularly memorable session was the one delivered by music therapy academic Simon Gilbertson. A year later, students recalled how this session had challenged their world view (Jennifer) or their understanding of research (Johnny). When analysing the interview transcripts, we wondered whether these challenges had come about from the teaching topic itself (music therapy with children with severe traumatic brain injuries) or Simon’s teaching methods, which promoted discussion and provoked new ways of thinking. This may emphasize the important influence of the teaching team and teaching methods when implementing medical humanities teaching in medical education. We were fortunate to have a small, engaged teaching team, who had strong interests in music and health, were experienced in different teaching approaches, and were well versed in educational theory. We were also teaching a small group of students, which allowed for detailed discussion and sharing of experiences. In order to achieve similar impacts with a larger cohort of students, a teaching team may wish to consider approaches such as peer-led or inquiry-based teaching.

If music is interwoven with medical students’ lives and has the potential to stimulate transformation, it makes sense that all students should have access to this opportunity for learning. Most medical students in our study expressed interest in including music in medical education, but doing so raises questions for curriculum developers. How could you teach a full cohort of 300 medical students about music and medicine in an already over-crowded curriculum? If music and medicine content was limited to one session, would this be enough? Our experience indicated that the format of a two week SSP allowed time for students to critique common assumptions about music and weigh up the value of music in everyday life. We doubt the same outcomes would be achieved if music was included as a one-off session, in which it is likely that music would be perceived as “edutainment” ( Bleakley, 2015, p. 47) or that students would feel they are not “musical enough” to benefit (in the words of one survey respondent). We would therefore be cautious about expecting music that is merely additive to realise the rich learning potential we observed.

McLellan et al. (2013) argued for inclusion of music in the “medical humanities component of undergraduate medical education” to complement “conventional disease-focused medicine” (pp. 177-78), while Bleakley (2015) advocated for greater integration of medical humanities in curricula as a “constructively critical intervention” (p. 50). Based on our research, we see potential for both an integrated and optional approach to including music in medical education. Music could be part of an integrated medical humanities programme in which the humanities support students to “adopt a more critical and reflective stance towards their work” ( Brody, 2011, p. 7). Music may be an ideal medium for promoting criticality among medical students, as it taps into their existing musical practices. Additionally, music may hold special appeal for students who identify as being musicians, who may be struggling to bring together seemingly different parts of themselves. For these students, extended music and medicine study may allow them to re-assess the value and purpose of music in their lives and to explore ways that music can be incorporated into their future medical careers. In our view, student selected components in music and medicine should remain available for these students with a strong interest in music. Rather than recommending a single approach to including music in the medical education curriculum, we propose that maximum educational benefit will be achieved through both core and optional provision.

### Limitations

This was a small scale exploratory study conducted at a single institution in the UK and was not intended to produce generalisable findings. Our music and medicine SSP was informed by the particular expertise of the teaching team and may not be representative of music and medicine teaching delivered elsewhere. Furthermore, our study was limited to students in the early years of their undergraduate medical education study and only a small number of enthusiastic students were interviewed about their experiences. It would therefore be interesting to explore the extent to which music remains important to medical students as they progress through the later years of the undergraduate course and on to postgraduate training.

## Conclusion

Findings from this study highlight potential benefits of studying music and medicine for medical students, including challenges to the way they use music in their own lives, the way they think about others, and promoting openness to new perspectives. We therefore propose that all students should have access to this learning, regardless of whether they expect music and medicine study to be beneficial. However, sessions on music and medicine may have particular relevance for students who are already interested in such topics. Our experience suggests that extended music and medicine study can expose students to potential career pathways that they had not considered before, one of the main drivers for the inclusion of student selected components in medical curricula ( General Medical Council, 1993). For this reason, we support the inclusion of music and medicine study as a student selected component in undergraduate medical courses, in addition to inclusion of music as part of integrated medical humanities teaching in medical education.

## Take Home Messages

• Medical students have high levels of music engagement and experience and recognise links between music and medicine
• Music and medicine study may support students’ development of critical thinking and openness to new perspectives
• Music and medicine study may have particular value for students who identify as musicians, who may question the place of music in their lives and perceive tensions between a musical identity and their developing identities as doctors
• Extended music and medicine study, such as student selected components, should be available to students with a strong interest in music

## Notes On Contributors

Dr Alison Ledger is Programme Lead for the intercalated medical education degree at the University of Leeds, UK. She supervises clinical education research at all levels, from undergraduate through to PhD. From 2000-2011, Alison worked as a music therapy practitioner, educator, and researcher in Australia and the Republic of Ireland and completed her doctorate on the topic of music therapy implementation in hospital settings.

Dr Viktoria Joynes is the Director of Studies and Senior Lecturer in Medical Education at the School of Medicine at the University of Liverpool, UK. As part of this role, she oversees new curriculum developments, quality assurance, student wellbeing and professionalism across all five years of the MBChB. Viktoria is a sociologist and qualitative researcher by background, and gained a PhD in Medical Education from the University of Leeds in 2014.
