# Supplementary material for: “A huge part of my life”: Exploring links between music, medical education, and students’ developing identities as doctors
Source: MedEdPublish (2016). 2018 Sep 3;7:183. [Version 1] doi: 10.15694/mep.2018.0000183.1 (PMC10701835; doi:10.15694/mep.2018.0000183.1)
Supplement: Peer review history

# Reviewer report: Reviewer response for version 1

**DOI:** 10.21956/mep.19666.r29361

**Published:** 2018-11-12

**Authors:** Martina Kelly

**Affiliations:** 1 University of Calgary

This review has been migrated. The reviewer awarded 4 stars out of 5 This paper reports a mixed quantitative (survey) and qualitative (interviews) evaluation of a special study module on music in medicine. The introduction is well written and builds an argument from the role of music in health, music in medicine and the gap of music in medical education. The authors used a previously published framework to scaffold their teaching philosophy and evaluation. They took a sensitive approach to data collection e.g. a colleague conducted the interviews with past students. Although their sample was relatively small, their analysis is thoughtful and likely of interest to colleagues thinking of running similar modules. It was interesting to see how many students engaged with music during the day-to-day, how many had learnt music and how this influenced their decision to take the course or not. The authors make some interesting observations on music and identity and how the course offered a means for students already interested in music to bring together two seemingly separate aspects of their identity. At a time when student burn out is so topical, the opportunity for students to continue to develop their interests seems sensible and potentially beneficial. Overall I enjoyed reading this paper and thank the authors for their engaging report.

Reviewer Expertise:

NA

No decision status is available

# Reviewer report: Reviewer response for version 1

**DOI:** 10.21956/mep.19666.r29360

**Published:** 2018-09-10

**Authors:** Subha Ramani

**Affiliations:** 1 Harvard Medical School, Brigham and Women's Hospital

This review has been migrated. The reviewer awarded 4 stars out of 5 This is an interesting paper on a novel approach to humanities in medicine- namely through music. The authors lay the foundation for why they believe music should be one of the humanities modalities used in medical education. They clearly describe the therapeutic effects of music therapy, particularly inclusion of music may enhance belonging and well-being. With all the attention on wellness in medicine, music may confer additional benefits in this arena. The authors also describe their areas of expertise which validates why this team has the skills to study this subject.- The introduction lays the groundwork for the study purpose and objectives, the reasons for including music in education.- The 2-week music and medicine project is very innovative in both its design and implementation as well as the emphasis on multi-disciplinary perspectives.- The methods section focuses on data sources and data collection only. Robust qualitative studies need the epistemological approach, justification of the approach, details of data analysis data and how rigor was ensured. All these details are missing.- Results: It would have been nice to see the actual survey. But I believe that the value of this paper is in its qualitative data rather than the numerical data, not sure if this is truly a mixed methods study since I see mostly qualitative results. I enjoyed reading the case studies with student quotes, creates a clear picture in my mind about these students.- There are very important points in the discussion section, but I wish the authors had given me a road map telling me where they are going, what key points they would be emphasizing and also their recommendations for areas of future research. They end with the need to include music as part of medical humanities training. The conclusions could be stronger and more emphatic. I really enjoyed reading this paper and following authors' recommendations. I would suggest that the writing could be more systematic (O'Brien et al paper in Academic Medicine on writing up qualitative studies) and the conclusion more emphatic.

Reviewer Expertise:

NA

No decision status is available

# Reviewer report: Reviewer response for version 1

**DOI:** 10.21956/mep.19666.r29359

**Published:** 2018-09-03

**Authors:** Trevor Gibbs

**Affiliations:** 1 AMEE

This review has been migrated. The reviewer awarded 5 stars out of 5 A vary interesting and slightly different paper in the Humanities theme. Instead of looking at a course that provided a subject to be taught, this paper looked at it from a very broad learning perspective- the result of which created some very interesting thoughts and observations. I particularly liked the authors' thoughts on how music and medicine was seen through a much more intrinsic lens- as a result of music there appear to be quantifiable and qualitative changes within the students perspective on health and perhaps a catalyst towards a change in their thoughts and potential actions.I would agree with the authors that a logical approach would be through having a course as an option rather than compulsory, given the fascinating reasons why students didn't choose the SSP. We need more courses such as these to open up the inner being of our students, and allow their free expression as to their futures.I would recommend this paper to anyone involved, not just in Humanities teaching, but those involved in all aspects of curriculum planning.

Reviewer Expertise:

NA

No decision status is available
